# Gallic acid inhibits Kaposi's Sarcoma‐associated herpesvirus lytic reactivation by suppressing RTA transcriptional activities

**DOI:** 10.1002/fsn3.2048

**Published:** 2020-12-03

**Authors:** Wen‐Ying Long, Guo‐hua Zhao, Yao Wu, Ying Liu

**Affiliations:** ^1^ Central Laboratory The Fourth Affiliated Hospital Zhejiang University School of Medicine N1 Shangcheng Avenue Yiwu 322000 China; ^2^ Department of Neurology The Fourth Affiliated Hospital Zhejiang University School of Medicine N1 Shangcheng Avenue Yiwu 322000 China

**Keywords:** apoptosis, gallic acid, KSHV, lytic activation, RTA

## Abstract

Kaposi's sarcoma‐associated herpesvirus (KSHV), an oncogenic virus, has two life cycle modes: the latent and lytic phases. KSHV lytic reactivation is known to be important both for viral propagation and for KSHV‐induced tumorigenesis. The KSHV replication and transcription activator (RTA) protein is essential for lytic reactivation. Gallic acid (GA), one of the most abundant phenolic acids in the plant kingdom, has been shown potential chemotherapeutic efficacy against microbial and cancer. However, the effects of GA on KSHV replication and KSHV‐induced tumorigenesis have not yet been reported. Here, we report that GA induces apoptotic cell death in BCBL‐1 cells in a dose‐dependent manner. GA inhibits KSHV reactivation and reduces the production of progeny virus from KSHV‐harboring cells. GA inhibits RTA transcriptional activities by suppressing its binding to target gene promoters. These results suggest that GA may represent a novel strategy for the treatment of KSHV infection and KSHV‐associated lymphomas.

## Graphical abstract

Gallic acid inhibits cell growth and induces cell apoptosis in PEL cells. Gallic acid inhibits KSHV lytic reactivation in KSHV‐infected cells. Gallic acid inhibits RTA transcriptional activities by modulating its binding to target gene promoters.

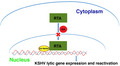

## 1 INTRODUCTION

Kaposi's sarcoma‐associated herpesvirus (KSHV) or human herpesvirus 8 (HHV‐8), a member of the γ‐herpesvirus family, is an oncogenic virus that has been causally linked to the development of three malignancies: Kaposi's sarcoma (KS) (Cai et al., 2010; Chang et al., 1994), primary effusion lymphoma (PEL) (Cesarman et al., 1995), and multicentric Castleman's disease (MCD) (Carbone et al., 2009; Gessain et al., 1996).

Similar to other herpesviruses, the replicative cycle of KSHV exists as latency and lytic replication (Jenner & Boshoff, 2002). KSHV mostly persists in the latent state during which it has a restricted latent gene expression program but can be reactivated and transitioned to the lytic state when triggered by stress conditions such as hypoxia or HIV coinfection, or stimulated by other chemical signals such as 12‐O‐tetradecanoylphorbol‐13‐acetate (TPA), sodium butyrate (NaB), and valproate (VPA) (Cai et al., 2006; Davis et al., 2001; Varthakavi et al., 1999).

During the lytic phase, the full spectrum of lytic viral genes (immediate‐early (IE), early (E), and late (L) genes), are sequentially expressed (Sun et al., 1999). The KSHV replication and transcription activator (RTA) is an IE gene that functions as a transcription factor to drive the temporally ordered expression of KSHV lytic genes leading to production of infectious viral particles; it activates many viral promoters, including those for PAN RNA, Ori‐Lyt‐associated RNA, ORF57, and K8, as well as its own promoter, by binding to RTA‐responsive elements (RREs) (Guito & Lukac, 2012; Lukac et al., 1999; Sun et al., 1998). KSHV lytic reactivation is known to be important both for viral propagation and for KSHV‐induced tumorigenesis (Purushothaman et al., 2015), so any interference that disrupts KSHV lytic switch could contribute to the treatment of KSHV‐related malignancies.

Gallic acid (GA) is one of the most abundant phenolic acids in the plant kingdom, with extensive application in the food and pharmaceutical industries (Fernandes & Salgado, 2016). Besides the edible uses of GA in the food industry, there are ample evidences that show the potential chemotherapeutic efficacy of GA against microbial, cancer, inflammatory, cardiovascular diseases, and neuropsychological diseases (Choubey et al., 2015). However, the effects of GA on KSHV replication and KSHV‐induced tumorigenesis have not yet been reported.

In this study, we investigated the antitumor and antiviral activity of gallic acid against human primary effusion lymphoma (PEL) cells. Our data demonstrate that GA induces apoptotic cell death in PEL cells. We also examined the effects of GA on KSHV replication and reactivation. GA inhibits RTA transcriptional activities via a mechanism involving modulating its binding to target gene promoters. These results suggest that GA may represent a novel strategy for the treatment of KSHV infection and KSHV‐associated lymphomas.

## 2 MATERIALS AND METHODS

### 2.1 Reagents

GA, VPA, NaB, TPA, and tetracycline were purchased from Sigma‐Aldrich Chemical Co. GA was dissolved in DMSO at 1 M as a stock solution. VPA and NaB were dissolved in sterile ddH_2_O at 1 M as a stock solution. TPA was dissolved at 200 μg/ml concentration with sterile ddH_2_O. Tetracycline was dissolved in DMSO at 1 mg/ml as a stock solution.

HRP‐conjugated goat anti‐mouse IgG and anti‐rabbit IgG were purchased from Santa Cruz Biotechnology. Mouse monoclonal antibody against Flag and rabbit monoclonal antibodies against GAPDH and tubulin were obtained from Cell Signaling Technology.

### 2.2 Cell culture and chemical treatment

iSLK.rKSHV.219 cells and HEK293T cells were cultured in DMEM (Gibco), and the body cavity‐based KSHV+ lymphoma cell line BCBL‐1 was maintained in RPMI1640 medium (Gibco). All these culture was supplemented with 10% fetal bovine serum (FBS) (Gibco) and 1% penicillin–streptomycin (Gibco).

iSLK.rKSHV.219 cells were pretreated with GA for 1 hr and then treated with 1 μg/ml tetracycline plus 1 mM valproate (VPA) (Sigma) for 48 hr to activate lytic replication. The BCBL‐1 cells were subcultured at 3 × 10^5^ cells/ml and pretreated after 24 hr with GA for 1 hr. Then, cells were treated with 20 ng/ml of 12‐O‐tetradecanoylphorbol‐13‐acetate (TPA) (Sigma) plus 0.3 mM NaB (Sigma) for 48 hr to activate lytic replication.

### 2.3 Cell viability assays

The effect of GA on PEL cell viability was determined by CCK‐8 assay. BCBL‐1 cells (1 × 10^4^ cells/well) were seeded onto 96‐well plates. After incubation for 2 hr, cells were treated with or without various concentrations of GA for 48 hr. Cell viability was determined by CCK‐8 assay. The untreated cells were utilized as control, and the cell viability was compared with the control. Each treatment was performed in triplicate, and three independent experiments were performed. Error bars represent the standard errors.

### 2.4 Two‐layered soft agar colony formation assay

For soft agar assay, experiments were carried out in 12‐well plates coated with a base layer of RPMI1640 containing 0.5% agar, 20% fetal bovine serum, and GA or DMSO. 5 × 10^3^ BCBL‐1 cells were seeded per well in RPMI1640 containing 0.35% agar, 20% fetal bovine serum, and GA or DMSO for 7 days. Colonies were visualized using a stereomicroscope (Olympus).

### 2.5 Quantitative reverse transcription‐PCR (qRT‐PCR)

Total RNA was extracted from cells using TRIzol (Life Technologies) according to the manufacturer's protocol. RNA was converted to cDNA by using RevertAid First Strand cDNA Synthesis Kit (Thermo) according to the manufacturer's protocol.

Relative transcript levels of selected cellular and genes were determined with gene‐specific primers plus SYBR^®^ Premix Ex Taq™Ⅱ (Tli RNaseH Plus) (TaKaRa) by 7,500 fast real‐time PCR system (Applied Biosystems). Primer sequences are listed in Table 1.

**Table 1:** Oligonucleotides used for PCR and qRT‐PCR analyses

Gene	Forward	Reverse
RTA	TATCCAGGAAGCGGTCTCAT	GGGTTAAAGGGGATGATGCT
Actin	GGGAAATCGTGCGTGACAT	GTCAGGCAGCTCGTAGCTCTT
ORF8.1	TGGTCGGCGGTTCAGTCATCAA	GCGGCCGCTAAGAAAATCGA
ORF59	TTAGAAGTGGAAGGTGTGCC	TCCTGGAGTCCGGTATAGAATC
ORF9	TAGGCGCTTCGTGCTGG	CCGGATTGCTGCACTCGTA
Bax	GACGGCAACTTCAACTGGG	CAGGGCCTTGAGCACCA
Caspase 9	CTGCGGCTGGTGGAAGAG	CTGCCCGCTGGATGTCCTC
Caspase 3	GCCTGTTCCATGAAGGCAG	CGTATGGAGAAATGGGCTGTAG
LANA	TCCAAAGTGTCAATGGAAGT	GTAGATGGGTCGTGAGAACA
Protrudin	GTCCTCCACCACCAGATGTT	TGAGGTCCTGGGAAGAGAGA
Ori‐Lyt	CCCTCCTTTGTTTTCCGGAAG	CTCATCGGGCCCTATTATAAAG
RTA promoter	GAACTACTCGAGCTGTGCCCTCCAGCTCTCAC	GGACGTAAGCTTACAGTATTCTCACAACAGAC

Relative expression levels were calculated using the ∆∆CT method after normalization to actin. Individual samples were assayed in duplication.

### 2.6 Quantitative analysis of KSHV virions in supernatant

Viral DNA was collected and prepared from culture supernatant by using the AxyPrep™ Body Fluid Viral DNA/RNA Miniprep Kit (Axygen) according to the manufacturer's protocol and then was used to amplify the KSHV LANA gene by qPCR using primers in Table 1.

Relative expression levels were calculated using the ∆∆CT method after normalization to protrudin (protrudin plasmid was added to supernatant as a control). Individual samples were assayed in duplication.

### 2.7 Luciferase reporter assay

Replication and transcription activator transactivation was quantified using the Dual‐Luciferase Reporter Assay System from Promega. Briefly, cells were transfected with indicated expression plasmids plus reporter plasmids. Cell lysates were prepared according to the manufacturer's protocol. Luciferase was measured on a GloMaxH‐Multi Microplate Multimode Reader (Promega). Data were taken as a ratio of firefly/Renilla luciferase, and the results shown represent experiments performed in duplicate.

### 2.8 ChIP assays

Cells were cross‐linked with 1% formaldehyde (Thermo Scientific) for 10 min at room temperature. Glycine was added to a final concentration of 125 mM to stop the cross‐linking reaction, and the samples were incubated for another 5 min at room temperature. Cells were washed twice with ice‐cold PBS and lysed in Fast ChIP buffer (50 mM Tris‐HCl [pH 7.5], 150 mM NaCl, 5 mM EDTA, 0.5 mM DTT, 0.5% IGEPAL CA‐630, 1.0% Triton X‐100) containing protease inhibitors. The extracted nuclei were pelleted by low‐speed centrifugation and resuspended in SDS lysis buffer (50 mM Tris‐HCl [pH 8.1], 10 mM EDTA, 1% SDS), and the samples were incubated on ice for 15 min. Lysates were sonicated and cleared by centrifugation for 30 min at 20,000 *g* and 4°C. Supernatants were combined with 9 volumes of ChIP dilution buffer (16.7 mM Tris‐HCl [pH 8.1], 167 mM NaCl, 1.2 mM EDTA, 1.1% Triton X‐100, 0.01% SDS) and subjected to immunoprecipitation for 12 hr at 4°C with gentle rotation using mouse anti‐Flag antibody or normal mouse IgG for HEK293T cells. Samples were centrifuged for 10 min at 20,000 *g*, 4°C, and supernatants were combined with 20 µl protein A/G beads (Thermo Scientific) and incubated for 3 hr at 4°C with gentle rotation. Immune complexes were washed with 1 ml each of low‐salt buffer (20 mM Tris‐HCl [pH 8.1], 150 mM NaCl, 2 mM EDTA, 1% Triton X‐100, 0.1% SDS), high‐salt buffer (20 mM Tris‐HCl [pH 8.1], 0.5 M NaCl, 2 mM EDTA, 1% Triton X‐100, 0.1% SDS), and LiCl buffer ( 10 mM Tris‐HCl [pH 8.1], 0.25 M LiCl, 1 mM EDTA, 1% IGEPAL CA‐630, 1% deoxycholic acid), and twice with TE buffer (10 mM Tris‐HCl [pH 8.1], 1 mM EDTA). To extract the DNA fragment, TE with 1% SDS, 0.2 M NaCl and protease K (Roche) were washed precipitates. After incubation at 65°C for 12 hr, the eluted solution was subjected to DNA gel extract kit (AXYGEN).

2 µl of 20 µl DNA solution was used as the template DNA for qPCR using primers specific for the A/T‐rich region of ori‐Lyt left and RTA promoter which were shown in Table 1.

### 2.9 Statistical analyses

All data are represented by the mean ± standard error of at least two independent experiments. Student's *t* test was used for statistical significance of the differences between treatment groups. Statistical analysis was performed using analysis of variance at 5% (*p* < .05).

## 3 RESULTS

### 3.1 GA inhibits cell growth and induces cell apoptosis in PEL cells

**FIGURE 1:**
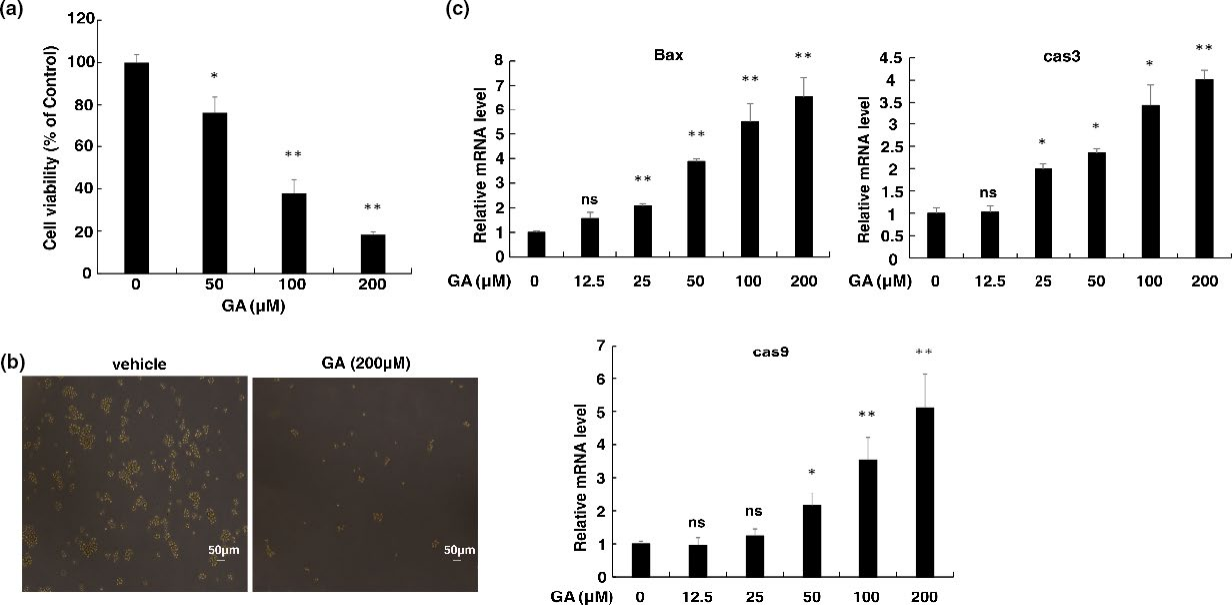
GA reduces cell viability and induces cell apoptosis in human herpesvirus 8 (HHV‐8)‐harboring primary effusion lymphoma (PEL) cells. (a) PEL cells (BCBL‐1) were treated with GA at the concentrations indicated for 48 hr; (b) soft agar colony formation assay. BCBL‐1 cells were cultured in soft agar containing 20% FBS and RPMI1640 with 200 μM GA for 7 days. (c) The mRNA level of BAX, Caspase 3, and Caspase 9 in BCBL‐1 cells treated with or without GA. Total RNA was extracted from BCBL‐1 cells, and purified RNA was subjected to RT‐PCR to detect mRNA expression of BAX, Caspase 3, and Caspase 9. The values obtained from untreated cells were defined as 1.0. All the data are presented as means of two technical replicates (*n* = 2, group values are indicated by mean ± *SEM*; *p < .05; **p < .01)

To determine the effects of GA on the PEL cells, a PEL cell line BCBL‐1 (Nador et al., 1996) (KSHV‐positive) was treated with 0, 50, 100, and 200 μM GA for 48 hr. The cellular viability was assessed using CCK‐8 assay. BCBL‐1 cells were susceptible to GA in a dose‐dependent manner (Figure 1a).

In addition, inhibitory effect of GA on colony formation of BCBL‐1 cells was elucidated. 200 μM GA significantly decreased colony formation of BCBL‐1 cells compared with DMSO‐treated cells (Figure 1b). Next, we examined whether the antiproliferative effects of GA are due to apoptotic cell death. BCBL‐1 cells were treated with GA at different concentrations for 48 hr, and the mRNA level of BAX, Caspase 3, and Caspase 9 were examined by RT‐PCR (Figure 1c). Results showed that the GA treatment increased the expression of BAX, Caspase 3, and Caspase 9.

As 200 μM GA caused most of the cell death, which was not conducive to the experiment. In the follow‐up experiments, we chose 100 μM as the highest concentration.

### 3.2 GA inhibits KSHV lytic reactivation in iSLK.rKSHV.219 cells

To determine whether GA functions in KSHV lytic replication, we selected the iSLK.rKSHV.219 cell line—harboring recombinant KSHV (rKSHV.219) with constitutive GFP expression that indicates latent infection and expressing RFP to report lytic reactivation upon lytic cycle induction (Vieira & O'Hearn, 2004)—as our experimental system.

**FIGURE 2:**
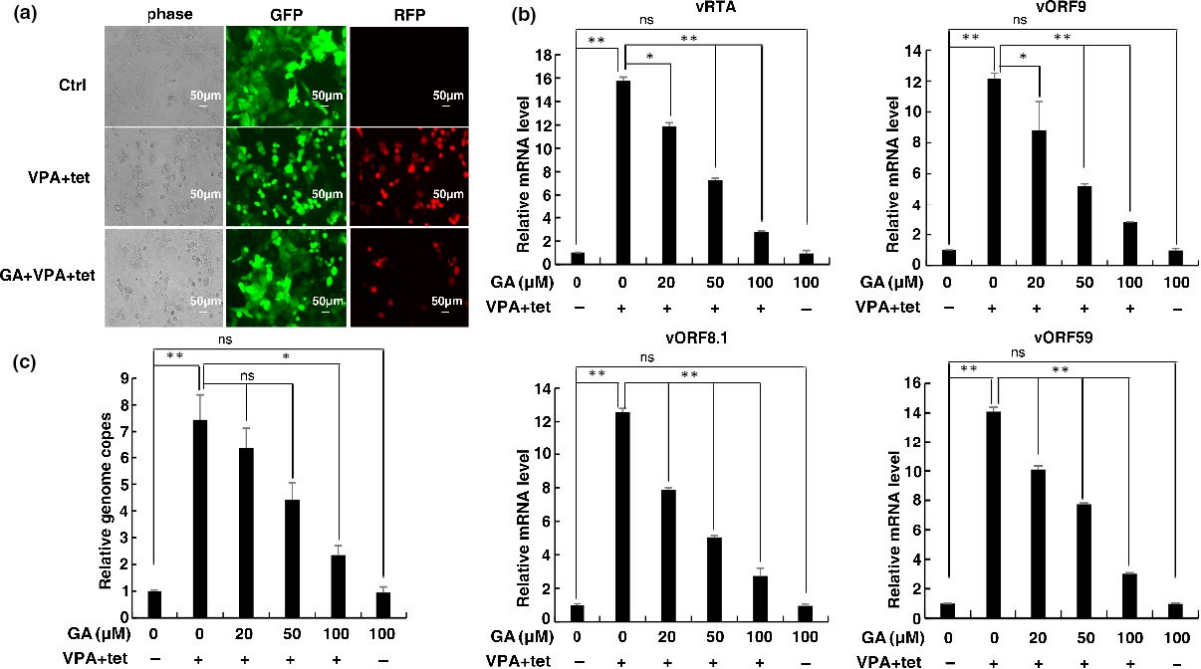
GA inhibits KSHV lytic reactivation in iSLK.rKSHV.219 cells. (a) iSLK.rKSHV.219 cells were treated with or without 100 μM GA for 1 hr followed by treatment with tetracycline (Tet) plus valproate (VPA) for 48 hr to induce KSHV lytic reactivation. The cells were photographed for GFP and RFP fluorescence. (b) iSLK.rKSHV.219 cells treated with GA at different concentrations for 1 hr followed by treatment with tetracycline (Tet) plus valproate (VPA) for 48 hr. mRNA levels of KSHV lytic genes RTA, ORF59, ORF9, and ORF8.1 were measured by RT‐qPCR, with normalization to actin using the ΔΔCT method (*n* = 2, group values are indicated by mean ± *SEM*; *p < .05; **p < .01). (c) Viral DNA in the media was quantified using qPCR, with normalization to an added plasmid protrudin using the ΔΔCT method. Data are presented as means of two technical replicates (*n* = 2, group values are indicated by mean ± *SEM*; *p < .05; **p < .01)

We treated iSLK.rKSHV219 cells with DMSO or 100 μM GA for 1 hr followed by treatment with tetracycline (Tet) plus valproate (VPA) for 48 hr to induce KSHV lytic reactivation. Photomicrographs showed there were a significant number of RFP+ cells in DMSO‐treated cells, but this was dramatically decreased in cells treated with GA (Figure 2a).

We next tested whether the inhibition effect of GA on KSHV lytic reactivation resulted from the inhibition of lytic gene expression. RT‐qPCR showed that, compared with DMSO‐treated iSLK.rKSHV.219 cells, GA‐treated cells had significant decreases in the mRNA levels of representative KSHV lytic genes RTA, ORF59, ORF9, and ORF8.1 (Figure 2b). ORF59 and ORF9 belong to KSHV early gene, when ORF8.1 belongs to KSHV late gene. As ORF59, ORF9, and ORF8.1 are downstream target genes of RTA, RTA can regulate their expression (Deng et al., 2007). Quantification of viral particles in the media revealed that cells treated with GA secreted fewer viral particles than those treated with DMSO (Figure 2c).

### 3.3 GA suppresses KSHV lytic reactivation in PEL cells

To determine whether GA functions in viral reactivation in other KSHV‐infected cells, we treated BCBL‐1 cells with 0, 20, 50, and 100 μM GA for 1 hr followed by treatment with NaB plus TPA to induce KSHV lytic reactivation.

**FIGURE 3:**
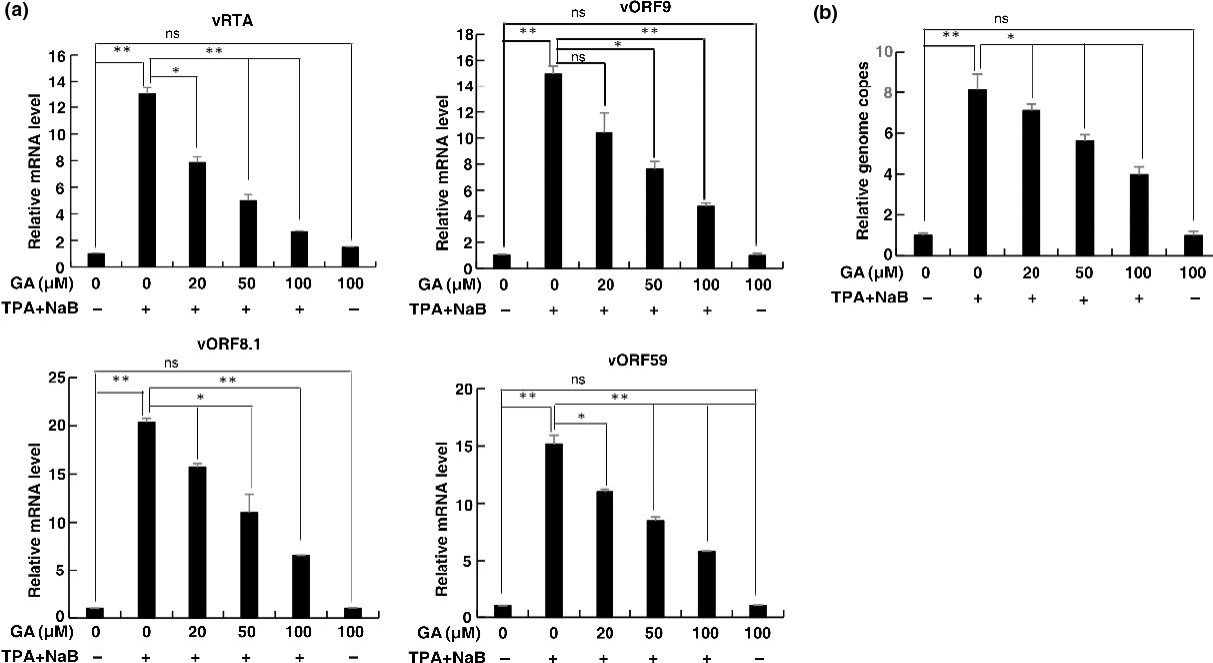
GA inhibits KSHV lytic reactivation in human herpesvirus 8 (HHV8)‐harboring primary effusion lymphoma (PEL) cells. (a) BCBL‐1 cells were treated with GA at different concentrations for 1 hr followed by treatment with TPA plus NaB for 48 hr. mRNA levels of KSHV lytic genes RTA, ORF59, ORF9, and ORF8.1 were measured by RT‐qPCR, with normalization to actin using the ΔΔCT method. (b) Viral DNA in the media was quantified using qPCR, with normalization to an added plasmid protrudin using the ΔΔCT method. Data are presented as means of two technical replicates (*n* = 2, group values are indicated by mean ± *SEM*; *p < .05; **p < .01)

RT‐qPCR revealed that the mRNA levels of the lytic genes RTA, ORF59, ORF9, and ORF8.1 were significantly decreased in GA‐treated cells in a dose‐dependent manner (Figure 3a). Furthermore, the decreases in these lytic gene expressions corresponded to decreases in the quantity of viral particles secreted into the culture media (Figure 3b).

### 3.4 GA inhibits RTA transcriptional activities by modulating its binding to target gene promoters

**FIGURE 4:**
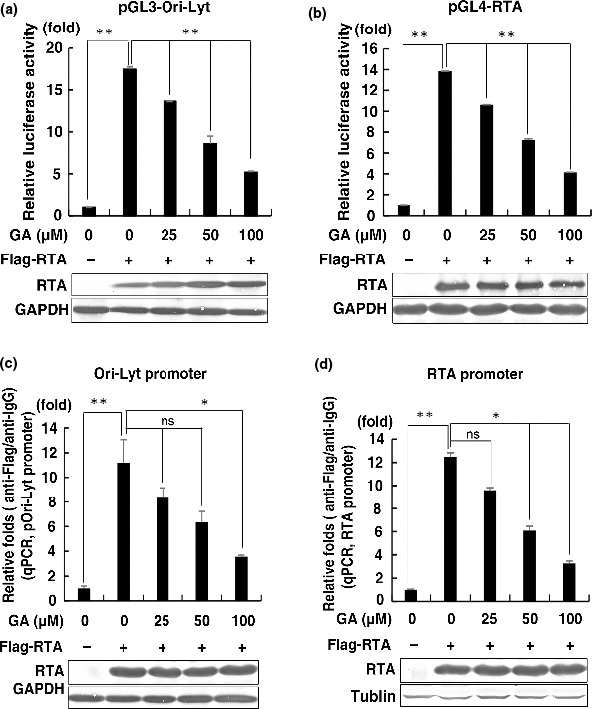
GA regulates the transcriptional activity of KSHV RTA by modulating its binding to the promoters of its target genes. GA inhibits RTA transcriptional activities in reporter assays. HEK293T cells were transfected with the firefly luciferase reporter constructs pGL3‐Ori‐Lyt‐Luc (a) or pGL4‐RTA‐Luc (b), a Renilla luciferase control plasmid, RTA, and treated with GA at different concentrations for reporter assay (*n* = 2, group values are indicated by mean ± *SEM*; *p < .05; **p < .01). GA inhibits RTA binding to the Ori‐Lyt promoter (c) and the RTA promoter (d). HEK293T cells were cotransfected with the reporter plasmid pOri‐Lyt‐Luc or pRTA‐Luc, with RTA‐Flag, and treated with GA at different concentrations. After 24 hr, the cells were harvested and subjected to ChIP assay using anti‐Flag magnetic agarose beads. The quantities of Ori‐Lyt promoter or RTA promoter DNA sequences in the precipitates were evaluated by qPCR using the ΔΔCT method. Data are presented as means of two technical replicates (*n* = 2, group values are indicated by mean ± *SEM*; *p < .05; **p < .01)

Given RTA is the known trigger for KSHV lytic activation (Lukac et al., 1998; West & Wood, 2003), we next examined whether GA would directly regulate RTA transcriptional activity by reporter gene assays. Expression of RTA increased the activities of the luciferase reporters pGL3‐Ori‐Lyt‐Luc and pGL4‐RTA‐Luc, but their activities were reduced when RTA transfected cells were treated with GA (Figure 4a,b).

To evaluate the mechanism underlying GA‐mediated inhibition of RTA transcription activity, we performed ChIP assays in HEK293T cells. In HEK293T cells transfected with RTA alongside reporter plasmids (pOri‐Lyt‐Luc or pGL4‐RTA‐Luc), GA decreased the interaction of RTA with both promoters of Ori‐lyt (Figure 4c) and RTA (Figure 4d) in a dose‐dependent manner. Since there showed no difference in RTA protein expression, we can exclude GA from inhibiting RTA expression to hinder the binding of RTA to their promoter region.

These results for reporter gene assay and ChIP together strongly suggest that the observed function of GA in regulating RTA's transcriptional activation of lytic genes results in some way to the modulation of RTA binding with its target gene promoters.

## 4 DISCUSSION

Gallic acid or 3, 4, 5‐trihydroxybenzoic acid (CAS No 149‐91‐7) is one of the most abundant phenolic acids in the plant kingdom (Al Zahrani et al., 2020). Besides the extensive application in the food and pharmaceutical industries, there are diverse scientific reporters on biological and pharmacological activities of GA (Akbari, (2020); Kahkeshani et al., 2019). There are many reports about the effects of GA on antimicrobial and cancer cell growth (Rajamanickam et al., 2018; Zhang et al., 2019). However, the effects of GA on KSHV and KSHV‐harboring cells have not been investigated.

In this study, we discovered that GA inhibits the proliferation of KSHV‐harboring PEL cells by inducing cell apoptosis. GA can inhibit the replication and reactivation of KSHV in KSHV‐harboring cells. Since KSHV is essential for KSHV‐infected tumors survival (Godfrey et al., 2005), we propose that GA leads to KSHV‐harboring cells death by inhibiting KSHV replication and reactivation. Through exploring the possible mechanism, we find that GA can inhibit RTA transcriptional activities by diminishing its binding to target gene promoters. These data indicate that GA may be a potential treatment for aggressive PEL and KSHV infection.

We have demonstrated that mRNA expression of lytic gene is suppressed by GA treatment in chemical reagents activated KSHV‐harboring cells; consistently, there were fewer viral particles in media from GA‐treated cells (Figures 2 and 3). From the mechanistic point of view, GA can inhibit motility, adherence, and biofilm formation of some microbial (Borges et al., 2012; Shao et al., 2015). So, it will be interesting to determine whether GA has an effect on KSHV de novo infection. As GA can reduce virus production, our next step will be to explore whether it also affects the infection efficiency of these viruses.

In our study, GA inhibits RTA transcriptional activities by diminishing its binding to target gene promoters (Figure 4). The binding of RTA and some target gene promoters is not directly, but at the same time, through the interaction with other host cell transcription factors (Liang & Ganem, 2004; Papp et al., 2019; Wang & Yuan, 2007). GA may affect the transcription activity of RTA by influencing other transcription factors. Thus, it will be fascinating to determine whether GA has an effect on these transcription factors which interact with RTA.

We determined that gallic acid inhibits the replication and reactivation of KSHV by repressing RTA transcriptional activities. Other herpesviruses also contain RTA or transcription factor similar to RTA (Walters et al., (2005); Walters et al., 2004; Goodwin et al., 2001), it will be interesting to determine whether GA can modulate other herpesviruses infection.

In conclusion, GA suppresses KSHV replication and reactivation, leading to apoptosis in KSHV‐harboring cells. These finding suggests that GA can be a potential anti‐KSHV drug candidate and may be considered as an effective treatment for KSHV‐related tumors.

## 6 CONFLICT OF INTEREST

The authors declare no conflict of interest.
